# Role of dual phase MDCT in renal cancer – beyond the renal mass

**DOI:** 10.1186/1470-7330-14-S1-P41

**Published:** 2014-10-09

**Authors:** D Santosh, D Fleming, M Robinson

**Affiliations:** 1 University Hospital Of Wales, Royal Gwent Hospital, Newport, UK

## Aim

To illustrate the anatomy of renal vasculature and its variants on cross-sectional imaging.

To highlight the benefits of obtaining images in both arterial and venous phase in staging and follow-up of renal cancer.

## Content

It is common practice to perform dual phase computed tomography (CT) in preliminary staging and subsequent follow-up of renal cancer patients in some institutions across the United Kingdom. We provide the best examples from our institution (2010-2013) with illustrations and the clinical relevance for the conditions stated below.

## Arterial phase

We discuss the normal anatomy and variants of the renal artery including early division of artery, accessory artery and double renal artery. In addition, usual and uncommon sites (e.g. muscle, small bowel, pancreas) of hypervascular metastasis in primary renal cancer patients will be illustrated.

## Portal-venous phase

We will highlight the normal anatomy and variants of the renal vein (e.g. aberrant, accessory renal veins) and associated tumour infiltration in unexpected veins (e.g. portal vein, gonadal vein) and solid organ metastasis.

## Conclusion

The renal vasculature is frequently visualised on imaging but often overlooked. This exhibit will provide radiology trainee’s an insight into the anatomical variants and its relevance in management of primary renal cancer. It reminds them of the common and uncommon metastasise and tumour infiltration seen in renal cancer, thus affecting the outcome.

